# Noninvasive Ambulatory Electrocardiographic Markers from Patients with COVID-19 Pneumonia: A Report of Three Cases

**DOI:** 10.3390/medicina60040655

**Published:** 2024-04-19

**Authors:** Motohiro Kimata, Kenichi Hashimoto, Naomi Harada, Yusuke Kawamura, Yoshifumi Kimizuka, Yuji Fujikura, Mayuko Kaneko, Nobuaki Kiriu, Yasumasa Sekine, Natsumi Iwabuchi, Tetsuro Kiyozumi, Akihiko Kawana, Susumu Matsukuma, Yuji Tanaka

**Affiliations:** 1 Department of General Medicine, National Defense Medical College, 3-2 Namiki, Tokorozawa 359-8513, Saitama, Japan; doc01083@ndmc.ac.jp (M.K.); cen361@ndmc.ac.jp (N.H.); yitanaka@ndmc.ac.jp (Y.T.); 2 Department of Integrative Physiology and Bio-Nano Medicine, National Defense Medical College, 3-2 Namiki, Tokorozawa 359-8513, Saitama, Japan; 3 Division of Infectious Diseases and Respiratory Medicine, Department of Internal Medicine, National Defense Medical College, 3-2 Namiki, Tokorozawa 359-8513, Saitama, Japan; ykimizuka@ndmc.ac.jp (Y.K.); con221@ndmc.ac.jp (Y.F.); kawana59@ndmc.ac.jp (A.K.); 4 Department of Traumatology and Critical Care Medicine, National Defense Medical College, 3-2 Namiki, Tokorozawa 359-8513, Saitama, Japan; kaneko5058@ndmc.ac.jp (M.K.); kiriu@ndmc.ac.jp (N.K.); ysekine99@ndmc.ac.jp (Y.S.); tkiyozumi@ndmc.ac.jp (T.K.); 5 Department of Laboratory Medicine, National Defense Medical College Hospital, National Defense Medical College, 3-2 Namiki, Tokorozawa 359-8513, Saitama, Japan; lab317@ndmc.ac.jp (N.I.); matsukuma@ndmc.ac.jp (S.M.); 6 Department of Defense Medicine, National Defense Medical College, 3-2 Namiki, Tokorozawa 359-8513, Saitama, Japan; 7 Department of Pathology and Laboratory Medicine, National Defense Medical College, 3-2 Namiki, Tokorozawa 359-8513, Saitama, Japan

**Keywords:** ambulatory electrocardiographic markers, coronavirus disease-19, COVID-19, global cardiac ischemia, Holter electrocardiograph, myocardial remodeling, SARS-CoV-2, sudden cardiac death

## Abstract

Coronavirus disease 2019 (COVID-19) has affected medical practice. More than 7,000,000 patients died worldwide after being infected with COVID-19; however, no specific laboratory markers have yet been established to predict death related to this disease. In contrast, electrocardiographic changes due to COVID-19 include QT prolongation and ST-T changes; however, there have not been studies on the ambulatory electrocardiographic markers of COVID-19. We encountered three patients diagnosed as having COVID-19 who did not have a prior history of significant structural heart diseases. All patients had abnormalities in ambulatory echocardiogram parameters detected by high-resolution 24 h electrocardiogram monitoring: positive late potentials (LPs) and T-wave alternans (TWA), abnormal heart rate variability (HRV), and heart rate turbulence (HRT). Case 1 involved a 78-year-old woman with a history of chronic kidney disease, Case 2 involved a 76-year-old man with hypertension and diabetes, and Case 3 involved a 67-year-old man with renal cancer, lung cancer, and diabetes. None of them had a prior history of significant structural heart disease. Although no significant consistent increases in clinical markers were observed, all three patients died, mainly because of respiratory failure with mild heart failure. The LP, TWA, HRV, and HRT were positive in all three cases with no significant structural cardiac disease at the initial phase of admission. The further accumulation of data regarding ambulatory electrocardiographic markers in patients with COVID-19 is needed. Depending on the accumulation of data, the LP, TWA, HRV, and HRT could be identified as potential risk factors for COVID-19 pneumonia in the early phase of admission.

## 1. Introduction

More than 7,000,000 patients have died from coronavirus disease 2019 (COVID-19) worldwide [1]. Acute myocardial damage, cardiac conduction disturbances, and chronic cardiovascular injuries, including thrombosis, can occur with COVID-19 [2,3]. The increased risk of cardiac death in patients with COVID-19 who may or may not have had previous cardiovascular diseases has also been reported [4]. Although risk stratification in the early stages of COVID-19 is important when considering therapeutic strategies, surrogate markers have not yet been established to predict prognosis. Changes in the electrocardiograms of patients with COVID-19 include QT prolongation, ST-T changes, and other repolarization abnormalities [5].

Recently, noninvasive 24 h Holter electrocardiograms have been used to measure electrocardiographic markers as predictors of sudden cardiac death or lethal arrhythmia in patients with cardiac diseases, including ischemic heart disease and heart failure [6,7,8]. Noninvasive electrocardiogram markers derived from ambulatory electrocardiogram devices indicate the late potential (LP), T-wave alternans (TWA), heart rate turbulence (HRT), and heart rate variability (HRV); these parameters can be measured simultaneously during a single sampling inspection to predict fatal arrhythmias or sudden cardiac death in patients with structural heart disease [6,7,8]. These parameters are known as ambulatory electrocardiographic markers (AECG-Ms). However, the usefulness of AECG-Ms in the early phases of treating COVID-19 pneumonia has been seldom reported. Herein, we report three cases in which all AECG-Ms were positive in patients with COVID-19.

## 2. Case Presentation

### 2.1. Case 1

A 78-year-old woman presented with coughing and dyspnea and was hospitalized with a diagnosis of COVID-19. She had a history of chronic kidney disease but no prior history of significant cardiovascular diseases. Her vital signs upon admission are shown in Table 1. QT prolongation and ST-T changes are not shown (Table 1).

To validate the usefulness of AECG-Ms in the acute phase of COVID-19, a high-resolution Holter electrocardiogram (1.5 V, 1000 Hz; Fukuda Denshi Co, Tokyo, Japan) was attached to the patient for 24 h within 48 h of admission, and the LPs, TWA, HRV, and HRT were measured. The LPs were determined as positive when at least two of the following three criteria were met: (1) a filtered QRS duration of >135 milliseconds, (2) a root-mean-square voltage of the signals of <20 μV in the last 40 milliseconds, and (3) a duration of >38 milliseconds of the low-amplitude signal after the voltage decreased to less than 40 μV [9]. The TWA value was determined to be positive when the reference value was exceeded; the TWA reference values were 19.9 μV when the noise level was less than 10 mV and 23.6 μV when the noise level remained between 10 and 20 mV [10]. For the HRV, the standard deviation of normal NN intervals was used as a parameter, and the cutoff value for poor prognosis was set as a standard deviation of normal NN intervals of <75 milliseconds [11]. For HRT, turbulence onset and turbulence slope were used as parameters, and a patient was determined to have abnormal HRT if the turbulence onset was ≥0% and the turbulence slope was ≤2.5 milliseconds per RR interval (Category 2) [8]. In addition, the cardiac function of the right and left ventricles was evaluated using cardiac ultrasonography. Procalcitonin and C-reactive protein levels were used as inflammatory markers. The soluble fibrin monomer complex, which is included in the evaluation of disseminated intravascular coagulation syndrome, was used as a coagulation marker. The laboratory data are summarized in Table 2. The echocardiographic findings are shown in Table 3.

**Figure 1:**
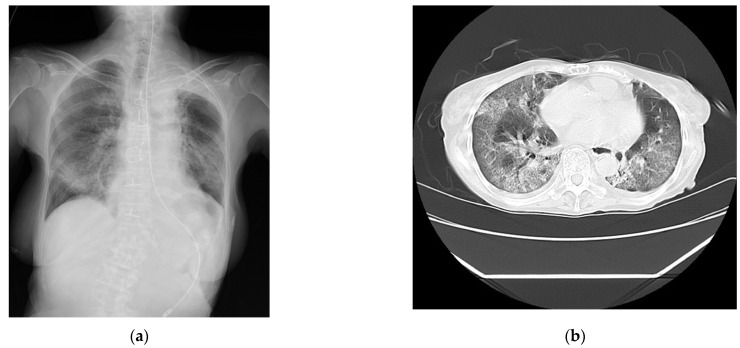

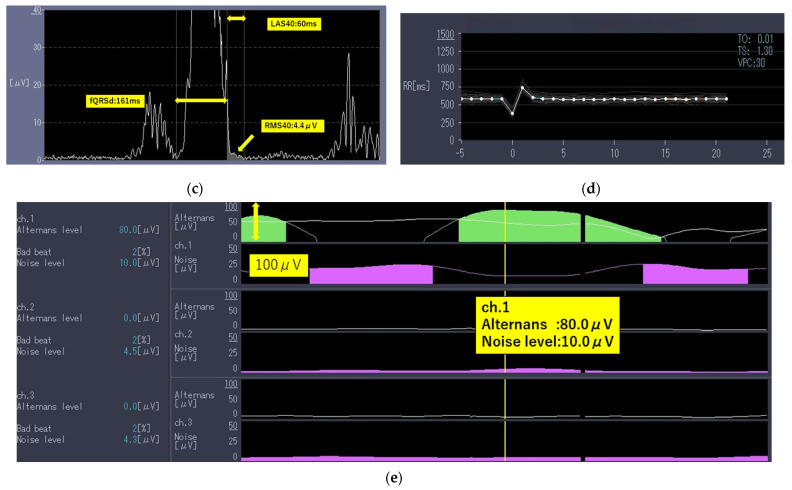
The findings for Case 1. (**a**) The chest radiograph captured on the day of admission. (**b**) The chest computed tomography image captured on the day of admission showing bilateral diffuse ground-glass opacities. (**c**) An analysis of the LPs measured using high-resolution 24 h Holter electrocardiography on the day after admission. The filtered QRS duration (fQRS) was 128 milliseconds, the root-mean-square voltage of the signals (RMS40) was 4.4 μV (<20 μV), and the duration of the low-amplitude signal after the voltage decreased to less than 40 μV (LAS40) was 60 milliseconds (>38 milliseconds). The patient was determined to be positive. (**d**) An analysis of the heart rate turbulence (HRT) measured using high-resolution 24 h Holter electrocardiography on the day after admission. The turbulence onset was ≥0%, and the turbulence slope was ≤2.5 milliseconds per RR interval. The patient was determined to have abnormal HRT (Category 2). (**e**) An analysis of T-wave alternans (TWA) measured using high-resolution 24 h Holter electrocardiography on the day after admission. Shaded in green area indicate the presence of TWA. The TWA was 80.0 μV with a noise level of 10.0 mV. The TWA reference value was 19.9 μV when the noise level was less than 10 mV. The patient was determined to be positive.

The laboratory test findings of Case 1 indicated that the inflammatory marker and brain natriuretic peptide levels were moderately to highly elevated. The chest radiographs (Figure 1a) and computed tomography images (Figure 1b) showed extensive ground-glass opacities and a crazy-paving pattern. The 24 h Holter electrocardiography performed upon admission showed that all AECG-Ms were positive (Table 4; Figure 1c–e).

The patient was treated with heparin at a dose of 10,000 U/day for 10 days, methylprednisolone at a dose of 1000 mg/day for 3 days, and dexamethasone at a dose of 6.6 mg/day for 5 days. She was not treated with any antiviral agents as she had poor renal function. The patient showed signs of improvement; however, an increase in the BNP levels from 178 pg/mL to 338 pg/mL, unexpected deterioration in renal function, electrolyte imbalance, and bleeding in the gastrointestinal tract were observed. She died of respiratory failure mainly because of pneumonia with heart failure preserved ejection fraction (HFpEF) on day 12 after admission. Just before the death of the patient, sustained ventricular tachycardia occurred with a decrease in O_2_ saturation; however, because of a DNAR (Do Not Attempt Resuscitation) statement provided by her family, resuscitation was not attempted. 

### 2.2. Case 2

**Figure 2:**
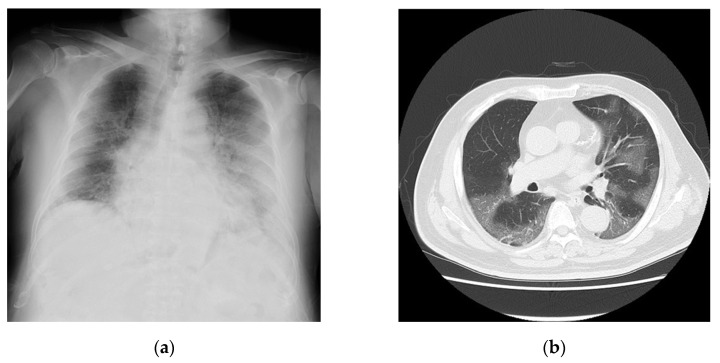
Imaging findings of Case 2 captured on day of admission. (**a**) Chest radiograph and (**b**) chest computed tomography image showing bilateral diffuse ground-glass opacities.

A 76-year-old man complaining of dyspnea was hospitalized after being diagnosed as having COVID-19 using a real-time polymerase chain reaction test. He had a history of hypertension, diabetes mellitus, and cerebral infarction but no history of significant cardiovascular diseases. His vital signs upon admission are shown in Table 1. QT prolongation and ST-T changes are not shown (Table 1). The laboratory data gathered are summarized in Table 2. The findings indicate that the inflammatory markers for this patient were moderately to highly elevated. The chest radiographs (Figure 2a) and computed tomography images (Figure 2b) reveal extensive ground-glass opacities. The echocardiographic findings are shown in Table 3. A 24 h Holter electrocardiography performed upon admission showed that all AECG-Ms were positive (Table 4). The patient was treated with heparin at a dose of 14,000 U/day for 12 days, remdesivir at a dose of 200 mg/day for 10 days, and dexamethasone at a dose of 6.6 mg/day for 10 days. However, his dyspnea worsened, the chest radiographs showed spreading ground-glass opacities, and he died from respiratory failure on day 12 after admission.

### 2.3. Case 3

**Figure 3:**
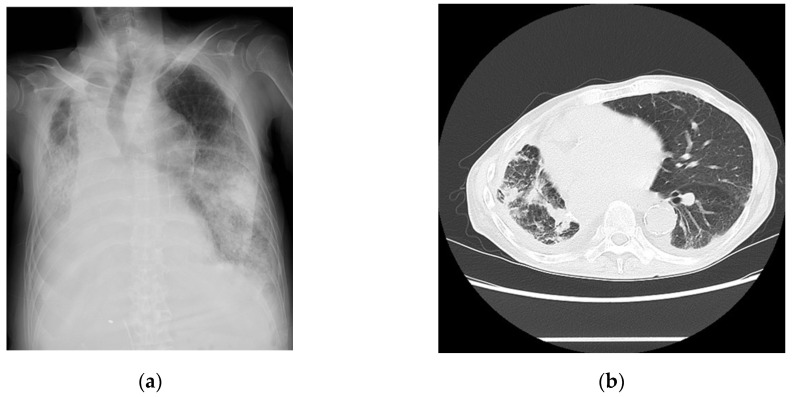
Imaging findings for Case 3 captured on day of admission. (**a**) Chest radiograph and (**b**) chest computed tomography image showing bilateral extensive findings of pneumonia with air bronchogram.

A 67-year-old man complaining of coughing and dyspnea was hospitalized with a diagnosis of COVID-19. He had a history of renal and lung cancers and pancreatic diabetes but no significant cardiovascular diseases. His vital signs upon admission are shown in Table 1. QT prolongation and ST-T changes are not shown (Table 1). The laboratory data are summarized in Table 2. An examination of the findings for this patient indicated that the levels of inflammatory markers were mildly elevated. The chest radiographs (Figure 3a) and computed tomography images (Figure 3b) revealed pneumonia. The echocardiographic findings are shown in Table 3. A 24 h Holter electrocardiography performed upon admission showed that all AECG-Ms were positive (Table 4). The patient was treated with heparin, remdesivir, and dexamethasone at a dose of 6.6 mg/day for 5 days. Oxygen saturation showed improvement on day 4 after admission, but the patient’s condition worsened from day 6, the BNP level increased from 162 pg/mL to 611 pg/mL, and carbon dioxide narcosis was induced. He was treated with methylprednisolone at a dose of 1000 mg/day for 3 days, remdesivir at a dose of 100 mg/day for 9 days, and heparin at a dose of 5000 U/day for 4 days, but he died from pneumonia with HFpEF on day 10 after admission.

## 3. Discussion

All three patients died from respiratory failure caused by COVID-19; in all three patients, the AECG-Ms—LPs, TWA, HRT, and HRV—were positive. In Cases 1 and 3, the patients were suspected of having comorbid HFpEF. In Case 2, there was a possibility that the patient had HFpEF. Although no remarkable consistent increases in the clinical markers were observed, there is a possibility that the AECG-Ms have the potential to be risk factors of COVID-19 pneumonia in the early phase of admission. AECG-Ms are reportedly useful in patients with structural cardiac diseases or chronic kidney disease; the markers could be used as predictors in cases where no obvious history of cardiac diseases is present at the time of admission to the hospital. The probability of each AECG-M being positive in the absence of cardiovascular diseases ranges from 5% to 10% [8,10,12,13]. Therefore, the probability of all four AECG-Ms being positive in a healthy heart is extremely low. Cases 1 and 3 had HFpEF at the time of admission, and heart failure worsened slightly during the clinical course. In contrast, Case 2 initially had no apparent heart failure, but HFpEF also progressed during the clinical course. HFpEF might be triggered by COVID-19 pneumonia. Notably, the AECG-Ms were positive for all parameters in these cases.

In patients who die because of COVID-19, the cardiovascular, immune, and coagulation mechanisms necessary to support life are disrupted approximately 3–4 weeks after viral exposure due to cytokine storms [14]. Consequently, the levels of cardiac biomarkers such as troponin and brain natriuretic peptide increase in patients with COVID-19 depending on disease severity. 

No obvious cardiac disease was initially noted in Case 2; however, a diabetic comorbidity with chronic kidney disease was present, and the risk of atherosclerosis seemed to be high. In all cases, systolic function was preserved, but increased BNP levels and/or cardio/thoracic ratio suggested the possibility of HFpEF at the time of UCG. However, the AECG-Ms—considered to be prognostic predictors of sudden cardiac death—were positive. We speculate that myocardial remodeling and ischemia were involved in these cases, as described below. Although no obvious cardiac disease was noted, Case 1 had a history of chronic kidney disease, and the AECG-Ms may have been useful for investigating cardiorenal syndrome. Hashimoto et al. reported that AECG-Ms are useful for predicting sudden cardiac death and fatal arrhythmias in patients with chronic kidney disease [15]; our findings for Case 1 are consistent with those of their study. Furthermore, Cases 1 and 3 also showed elevated BNP levels, suggesting the presence of HFpEF. In contrast, surprisingly, the AECG-Ms were positive for all items in Case 2, even though there was no initial sign of obvious cardiac disease. In COVID-19 pneumonia, AECG-Ms might have the potential to predict sudden cardiac death in patients without obvious cardiac disease.

In the cases reported here, we analyzed four specific parameters: LPs, TWA, HRV, and HRT. These markers have been reported to be predictive of fatal arrhythmias and/or cardiac death in patients with both cardiac and chronic kidney diseases [6]. All AECG-Ms have been reported to be highly useful, especially for treating ischemic heart disease [16,17,18,19]. LPs and TWA are more likely to be positive for remodeling resulting from cardiac diseases. LPs reflect a conduction delay at sites of myocardial degeneration between the necrotic and healthy myocardia; they are useful for predicting the occurrence of lethal ventricular arrhythmias because conduction delays can be electrical substrates for VT, such as myocardial infarction and cardiomyopathy. Determining TWA is a well-established method for testing beat-to-beat T-wave amplitude alternations at the microvolt level and is thought to reflect repolarization abnormalities that lead to fetal arrhythmias. Similar to LPs, TWA is particularly useful in predicting lethal ventricular arrhythmias and sudden cardiac death in patients with ischemic heart disease. Tondas et al. reported that the TWA levels are elevated at discharge compared with those at admission in patients with COVID-19 [20]. HRV evaluates autonomic function based on variability in RR intervals. Many studies have revealed that patients with a small variability have poor prognoses. In patents with COVID-19, HRV alone has been described as useful for predicting prognosis [21]. The HRT measures acceleration in the early phase and deceleration in the late phase of the RR interval derived from baroreceptor reflexes after ventricular extrasystoles to assess the autonomic imbalance associated with poor prognosis, especially in ischemic heart disease.

In the current study, the AECG-Ms were positive in patients with COVID-19 who did not show prominent abnormalities in the inflammatory and coagulation laboratory and UCG markers or who had a history of cardiac disease. We speculate that two pathophysiological considerations may support these findings: (1) Severe acute respiratory syndrome coronavirus 2 may cause angiotensin-converting enzyme 2 receptor-mediated myocardial remodeling. (2) Global myocardial ischemia is caused by hypoxemia due to spasms and thrombus formation. 

Regarding myocardial remodeling, the expression of angiotensin-converting enzyme 2 receptors decreases in conjunction with that of intramyocardial angiotensin-converting enzyme 2 receptors and viral spike protein, and then angiotensin 2 expression increases through positive feedback. This, in turn, activates ADAM17, which activates TNF-α, thereby triggering inflammation. This mechanism is thought to cause progressive remodeling of myocardial tissues, resulting in electrophysiological substrate abnormalities. 

Global myocardial ischemia may play an important role as a modifier. Unlike localized ischemia, such as in coronary artery disease, global myocardial ischemia may present as a diffuse myocardial injury caused by various mechanisms. Myocardial ischemia in patients with COVID-19 may be caused by (1) an oxygen supply-and-demand imbalance caused by pneumonia, (2) a vascular spasm due to immune cell activation, and (3) plaque disruption and embolization due to virus-induced endothelial cell injury [22]. 

The direct cause of death in all three cases in this study was mainly respiratory failure due to severe pneumonia and comorbidities with HFpEF. The AECG-Ms, markers for cardiac death, were positive in all cases. The reason for this is that in severe COVID-19 cases, myocardial remodeling and global myocardial ischemia progress in parallel with respiratory failure; although patients often eventually die from respiratory failure, myocardial damage may also occur. ACE2 receptors are not only distributed in the respiratory system, but also in the heart and other organs, and patients with COVID-19 pneumonia may suffer simultaneous multiorgan injury from cytokine storms. The primary cause of death in these cases was respiratory failure; however, cardiac comorbidities were also present. More specifically, Case 1 exhibited comorbid HFpEF and developed sustained ventricular tachycardia just before death. In Case 3, >1000 premature ventricular contractions per 24 h were observed; these could be a trigger for lethal arrythmia, which did not occur. In patients with COVID-19 pneumonia, ACE2 receptor-mediated myocardial cell remodeling and global micro-myocardial ischemia may have been suggested. We hypothesized that these effects may lead to the appearance of electrophysiological abnormalities at the cellular level, which may be reflected in the AECG-Ms in the early phases of hospitalization. The markers may acutely reflect a condition that is becoming more severe because of a cytokine storm at an earlier stage; however, we only report on three cases here, and the usefulness of AECG-Ms should be examined in studies with large populations.

The phenomenon of all four AECG-M being positive is unusual in patients without a prior history of a significant cardiac disease. Therefore, COVID-19 might cause electrophysiological modifications as a result of HFpEF. Further studies are needed to accumulate more data and statistically determine the usefulness of the AECG-Ms.

This study had some limitations. First, we could not evaluate tissue Doppler using UCG because the UCG equipment at the COVID-19 department of our hospital did not have the tissue Doppler function. Furthermore, we could not measure the *E*/*e*’, and therefore, the precise diastolic function was unknown for the three cases. However, apparently, the systolic function was preserved in all three cases, and the BNP level progressively increased during the clinical course, especially in Cases 1 and 3. Therefore, the cases could have been affected by HFpEF. In addition, because of facility limitations in the COVID-19 ward at our institution, no imaging studies other than ultrasonography were performed to investigate myocardial abnormalities. Second, although the patients in this study had no diagnosed heart disease at the time of admission, they had cardiovascular risk due to chronic renal failure, stroke, and a history of diabetes. Therefore, further investigation is needed to determine whether the results of this study are valid for patients with no risk factors for cardiac disease. However, the findings of this case report are meaningful because all patients without abnormalities on 12-lead ECG at admission showed positive AECG-Ms, and they can provide preliminary data for clinical studies in the future. Third, none of the drugs excluding remdesivir were reported to cause AECG-M abnormalities. Regarding remdesivir, the possibility that the drugs used may have affected the AECG-Ms cannot be ruled out completely. A positive correlation between remdesivir and AECG-M has not been reported. Although QT prolongation has been reported with remdesivir [23,24], there were no findings of QT prolongation in Cases 2 and 3 in which the patients received remdesivir. All patients who had all positive A-ECG markers died; therefore, we speculate that there is a low possibility that all positive AECG-Ms are not only affected by drugs such as remdesivir.

## 4. Conclusions

Although AECG-Ms are prognostic markers for cardiac death in patients with apparent heart diseases, all of these markers were positive in all three patients who had HFpEF and died from respiratory failure due to COVID-19. If the clinical utility of the AECG-Ms is made evident through studies using a large number of patients with COVID-19, the markers may be used as early diagnostic tools to determine whether a patient should be critically managed.

## Figures and Tables

**Table 1:** A summary of the demographic characteristics and clinical features of the patients in the **three cases**.

Characteristic	Case 1	Case 2	Case 3
Age (years), sex	78, female	76, male	67, male
Race	Asian	Asian	Asian
Prior history	Myasthenia gravis, postoperative phrenic nerve injury, and chronic kidney disease	Hypertension, diabetes mellitus, and cerebral infarction	Renal cancer, lung cancer, and pancreatic diabetes
Medication for cardiac diseases	−	−	−
Significant structured heart disease	−	−	−
Medication for hypertension/diabetes/dyslipidemia	−	DPP-4 inhibitor, SGLT2 inhibitor, and metformin	Insulin
Family history of sudden cardiac death	−	−	−
History of smoking	−	+	+
Vital signs upon admission			
Blood pressure, mmHg	122/73	116/91	177/97
Pulse rate, beats per minute	107	89	96
Body temperature, °C	38.1	37.0	36.9
Respiratory rate, breaths per minute	34	20	23
Oxygen saturation, %	95 (with oxygen mask and reservoir bag, 8 L/min flow)	95 (with oxygen mask, 4 L/min flow)	96 (with oxygen mask, 3 L/min flow)
12-lead electrocardiogram			
QT prolongation	−	−	−
QT, millisecond	352	382	427
QTc, millisecond	406	384	432
ST-T changes	−	−	−
Arrhythmia throughout hospitalization			
Atrial fibrillation	None	None	None
PSVT	None	None	None
Atrial tachycardia	None	None	None
NSVT	None	None	None
SVT	Positive	None	None
PVC > 1000/24 h	None	None	Positive

Abbreviations: DPP-4, dipeptidyl peptidase-4; SGLT2, sodium–glucose cotransporter 2; PSVT, paroxysmal supraventricular tachycardia; NSVT, non-sustained ventricular tachycardia; SVT, sustained ventricular tachycardia; PVC, premature ventricular contraction.

**Table 2:** Laboratory findings for each case.

Finding	Case 1	Case 2	Case 3
White blood cell count, μL	12,000	10,900	7100
Hemoglobin, g/dL	10.9	17.2	10.3
Platelet count × 10^4^/μL	23.3	10.2	23.9
AST, U/L	20	44	19
ALT, U/L	9	36	8
LDH, U/L	251	417	137
Total protein, g/dL	6.1	6.4	7.0
Albumin, g/dL	3.1	3.1	2.0
BUN, mg/dL	38	30	14
Creatinine, mg/dL	1.53	1.11	0.57
eGFR, mL/min	25.8	56.6	107.3
Procalcitonin, ng/dL	0.3	0.4	0.17
CRP, mg/dL	19.7	20.3	6.5
BNP, pg/mL	178.3	20.3	162.0
Troponin I, pg/mL (reference value: <28.0)	272.8	10.6	N/A
D-dimer, ng/mL	1.0	1.8	3.5
SF, μg/mL (reference value: <3.0)	33.3	26.9	N/A

Abbreviations: ALT, alanine aminotransferase; AST, aspartate aminotransferase; BNP, brain natriuretic peptide; BUN, blood urea nitrogen; eGFR, estimated glomerular filtration rate; CRP, C-reactive protein; LDH, lactate dehydrogenase; SF, soluble fibrin.

**Table 3:** Echocardiographic findings of each case.

Finding	Case 1	Case 2	Case 3
IVS, mm	8.5	7.0	9.3
LVDd, mm	35.6	51.0	50.5
PWT, mm	10.3	10.2	8.7
LVEF, %	82.7	75.1	64.2
RVFAC, %	44.4	53.9	−
TAPSE, mm	20.5	22.7	−

Abbreviations: IVS, interventricular septum; LVDd, left ventricular end-diastolic diameter; LVEF, left ventricular ejection fraction; PWT, posterior left ventricular wall thickness; RVFAC, right ventricular fractional area change, TAPSE, tricuspid annular plane systolic excursion.

**Table 4:** Data for ambulatory electrocardiographic markers and arrhythmia.

Marker	Abnormal Value or Cutoff Value	Case 1	Case 2	Case 3
Late potential				
fQRS, millisecond	>135	128	136	160
RMS40, μV	<20	4.4	1.5	10.4
LAS40, millisecond	>38	60	53	57
Determination		Positive	Positive	Positive
T-wave alternans				
Noise level, mV	(*1), (*2)	10.0	9.6	12.3
TWA, μV	(*1), (*2)	80.0	28.4	35.7
Determination		Positive	Positive	Positive
Heart rate variability				
SDNN, millisecond	<75	41.6	64.0	64.0
Determination		Positive	Positive	Positive
Heart rate turbulence				
TO, %	≥0	0.01	0.01	0.01
TS, millisecond/RR interval	≤2.5	1.30	1.00	1.00
Determination		Abnormal (Category 2)	Abnormal (Category 2)	Abnormal (Category 2)

Abbreviations: fQRS, filtered QRS duration; LAS40, duration of low-amplitude signal after voltage decreased to <40 μV; RMS40, root-mean-square voltage of signals in last 40 milliseconds; SDNN, standard deviation of normal RR intervals; TO, turbulence onset; TS, turbulence slope. (*1) TWA reference value was 19.9 μV when noise level was less than 10 mV. (*2) TWA reference value was 23.3 μV when noise level remained between 10 and 20 mV. For Category 2, the turbulence onset was ≥0%, and the turbulence slope was ≤2.5 milliseconds per RR interval.

## Data Availability

The data presented in this study are available upon request from the corresponding author.
